# Telomere stabilization by metformin mitigates the progression of atherosclerosis via the AMPK-dependent p-PGC-1α pathway

**DOI:** 10.1038/s12276-024-01297-w

**Published:** 2024-09-02

**Authors:** Jin Young Sung, Seul Gi Kim, So-Young Park, Jae-Ryong Kim, Hyoung Chul Choi

**Affiliations:** 1 Department of Pharmacology, College of Medicine, Yeungnam University, 170 Hyunchung-Ro, Nam-Gu, Daegu, 42415 Republic of Korea; 2 Senotherapy-based Metabolic Disease Control Research Center, College of Medicine, Yeungnam University, 170 Hyunchung-Ro, Nam-Gu, Daegu, 42415 Republic of Korea; 3 Department of Physiology, College of Medicine, Yeungnam University, 170 Hyunchung-Ro, Nam-Gu, Daegu, 42415 Republic of Korea; 4 Department of Biochemistry and Molecular Biology, College of Medicine, Yeungnam University, 170 Hyunchung-Ro, Nam-Gu, Daegu, 42415 Republic of Korea

**Keywords:** Dyslipidaemias, Dyslipidaemias

## Abstract

Telomere dysfunction is a well-known molecular trigger of senescence and has been associated with various age-related diseases, including atherosclerosis. However, the mechanisms involved have not yet been elucidated, and the extent to which telomeres contribute to atherosclerosis is unknown. Therefore, we investigated the mechanism of metformin-induced telomere stabilization and the ability of metformin to inhibit vascular smooth muscle cell (VSMC) senescence caused by advanced atherosclerosis. The present study revealed that metformin inhibited the phenotypes of atherosclerosis and senescence in VSMCs. Metformin increased the phosphorylation of AMPK-dependent PGC-1α and thus increased telomerase activity and the protein level of TERT in OA-treated VSMCs. Mechanistically, the phosphorylation of AMPK and PGC-1α by metformin not only enhanced telomere function but also increased the protein level of TERT, whereas TERT knockdown accelerated the development of atherosclerosis and senescent phenotypes in OA-treated VSMCs regardless of metformin treatment. Furthermore, the in vivo results showed that metformin attenuated the formation of atherosclerotic plaque markers in the aortas of HFD-fed ApoE KO mice. Although metformin did not reduce plaque size, it inhibited the phosphorylation of the AMPK/PGC-1α/TERT signaling cascade, which is associated with the maintenance and progression of plaque formation, in HFD-fed ApoE KO mice. Accordingly, metformin inhibited atherosclerosis-associated phenotypes in vitro and in vivo. These observations show that the enhancement of telomere function by metformin is involved in specific signaling pathways during the progression of atherosclerosis. These findings suggest that telomere stabilization by metformin via the AMPK/p-PGC-1α pathway might provide a strategy for developing therapeutics against vascular diseases such as atherosclerosis.

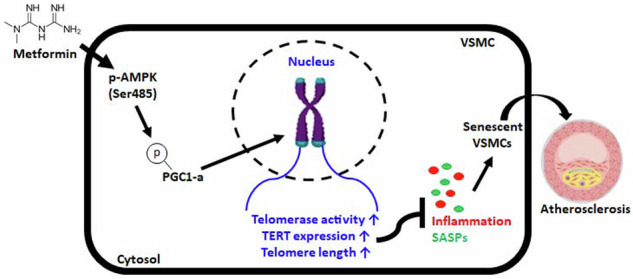

## Telomere stabilization with metformin: a novel approach to atherosclerosis

Atherosclerosis is a condition where fats build up in arteries, causing heart disease. A study investigates the effect of Metformin, a diabetes drug, on this condition. Researchers studied how Metformin affects the aging of vascular smooth muscle cells. The study used cell cultures and mice to examine Metformin’s effect on cell aging and atherosclerosis. The experiment involved treating cells and mice with Metformin and observing changes in inflammation, plaque formation, and cell aging. The findings showed that Metformin slows down aging in VSMCs and reduces plaque formation in mice, indicating it might be useful beyond diabetes treatment. The study concludes that Metformin’s ability to enhance cell health and lessen atherosclerosis could be due to its impact on cell aging processes. This finding paves the way for using Metformin to prevent heart disease.

This summary was initially drafted using artificial intelligence, then revised and fact-checked by the author.

## Introduction

Atherosclerosis is a condition characterized by the accumulation of fatty deposits, inflammation, and the formation of plaques within arteries. Vascular smooth muscle cells (VSMCs) play a crucial role in the development and progression of atherosclerosis^1^ and are the primary cells in the walls of all blood vessels. However, during the development of atherosclerosis, VSMCs can undergo cellular senescence, and in this disease context, senescent VSMCs are implicated in various pathological processes, such as reduced elasticity^2^, the promotion of proinflammatory molecules^3^, and matrix remodeling^4^. Understanding the mechanisms of VSMC senescence in atherosclerosis is important for developing strategies to mitigate its effects and potentially prevent or treat this disease.

Telomeres are protective caps at the ends of chromosomes that shorten as cells divide and age^5^ and play a crucial role in maintaining genomic stability. However, they are also associated with the aging process and various age-related diseases, including atherosclerosis^6^. Furthermore, shortening telomeres can lead to cellular senescence, a state in which cells lose their ability to divide and function properly^7^, and senescent cells can accumulate in atherosclerotic plaques and promote inflammation, thus contributing to plaque formation and instability^8^. In addition, telomere shortening is associated with accelerated biological aging and thus with increased risks of age-related diseases, including atherosclerosis^9^. Nonetheless, while there is evidence suggesting a link between telomere length and atherosclerosis, this relationship is complex and influenced by various factors, including genetic and lifestyle factors. Currently, the mechanisms linking telomeres and atherosclerosis are the subject of active research.

Metformin (Met) is commonly prescribed for type 2 diabetes^10^, but in recent years, it has attracted increased attention due to its potential effects on the aging process and age-related diseases, which include improved glucose metabolism, reduced inflammation, and protection against age-related conditions, such as cardiovascular disease^11^. Met also affects various metabolic pathways, including the AMP-activated protein kinase (AMPK) pathway, which is involved in energy regulation and cellular metabolism, and these effects are thought to play a role in the potential anti-aging effects of Met^12^. In addition, some studies have reported that Met can activate AMPK, a cellular energy sensor^13^, and that this activation can influence PGC-1α (peroxisome proliferator-activated receptor gamma coactivator 1-alpha) and its downstream pathways, leading to enhanced mitochondrial function and increased energy expenditure^14^. The AMPK signaling pathway controls the aging process through several signaling networks and different phosphorylation sites. AMPK is generally activated by phosphorylation at Thr172, as indicated by an elevated AMP/ATP ratio, and phosphorylation of AMPK at Ser485 has been identified as another phosphorylation site that is targeted by the AKT pathway^15^ and regulates lipid metabolism^16^. Although Thr172 is well known as the main phosphorylation site on AMPK, the activity of Thr172 and Ser485 have the same effect on cellular senescence. A previous study demonstrated that the phosphorylation of AMPK at Ser485 alleviates the senescence of VSMCs^17^. Therefore, similar to Thr172, Ser485 is a phosphorylation site involved in the anti-aging and anti-atherosclerosis effects of AMPK. On the other hand, PGC-1α is a transcriptional coactivator and a critical regulator of mitochondrial biogenesis, oxidative metabolism, and various metabolic processes^18^, and some studies have suggested that Met may indirectly affect PGC-1α and its related pathways to benefit metabolic health. However, the mechanism by which Met interacts with PGC-1α is unclear, and this topic remains an active area of investigation. Notably, this interaction may contribute to the anti-aging and anti-atherosclerotic effects of Met by improving telomere function through an AMPK-dependent p-PGC-1α pathway. Here, we report that metformin strongly protects telomere function during aging-associated atherosclerosis.

## Materials and methods

### Primary cell culture

Sprague‒Dawley rats (8-week-old males) were euthanized with 95% CO_2_, and VSMCs isolated from thoracic aortas were transferred to a culture dish containing cold (4 °C) Dulbecco’s modified Eagle’s medium (DMEM; WELGENE, Gyeongsangbuk-do, South Korea). After the fat tissue was removed, the arteries were cut longitudinally, and the intima was softly rubbed with cotton swabs to remove endothelial cells. Media were cut into ~1 mm squares and transferred to cell culture plates. DMEM containing 20% FBS/1% antibiotics was carefully added, and tissue blocks were incubated in a CO_2_ incubator (5% CO_2_/95% air) at 37 °C undisturbed for 5 days. The cells were grown to confluence, and VSMCs at passages 3 to 7 were used for the experiments. Metformin (Met) was obtained from Sigma (St. Louis, MO, USA) and dissolved in distilled water (DW).

### Western blotting

Western blotting was conducted as we previously described^19^. The blots were first incubated with the following proteins: anti-TERT (#37751 for Western blotting), anti-p53 (#126), anti-TNF-α (#133192), anti-ADRP (#377429), anti-Elastin (#58756), anti-p-Ser (#81514), anti-PGC-1α (#517380), 53BP1 (#515841), anti-IL-6 (#28343), anti-IL-10 (#365858), and anti-β-actin (#5867) antibodies obtained from Santa Cruz Biotechnology (Dallas, TX, USA). Anti-p-AMPK (Thr172) (#2535), anti-p-AMPK (Ser485) (#4185), anti-AMPK (#2532), and anti-MMP-2 (#87809) antibodies were obtained from Cell Signaling Technology (Beverly, MA, USA). Anti-collagen I (#260043) and anti-α-smooth muscle actin (SMA) (#7817) antibodies were obtained from Abcam (Cambridge, UK). The anti-TERT (#600-401-252, for immunofluorescence analysis) antibody was obtained from Rockland Immunochemicals, Inc. (Bridgeport, NJ, USA).

### Labeling with the BODIPY probe

To assess lipid accumulation, VSMCs and mouse tissues were stained with 2 μM BODIPY (493/503 nm) lipid probe (Thermo Fisher Scientific, Inc., MA, USA) in serum-free medium, incubated for 30 min at 37 °C in the dark, washed three times with PBS, and stained with DAPI (Invitrogen, Carlsbad, CA, USA) or α-SMA. Increases in green fluorescence intensity were observed in the FITC channel. The images were observed under a K1-Fluo confocal laser scanning microscope (Nanoscope Systems, Daejeon, Korea).

### SAβG staining of cells and tissues

SAβG staining of VSMCs and the en face of the aortas was performed as previously described^20^. SAβG activity was determined by X-gal staining (Thermo Fisher Scientific, Inc., MA, USA) according to the manufacturer’s instructions.

### Immunofluorescence staining of cells and tissues

VSMCs were fixed in 4% paraformaldehyde for 30 min, permeabilized with 0.2% Triton X-100 for 5 min at room temperature (RT), washed with PBS, blocked with 1% bovine serum albumin (BSA) in 1× PBS, and incubated with primary antibodies against collagen I, elastin, TERT, 53BP1, MMP-2, IL-6, and IL-10 (1:200) overnight at 4 °C. The cells were then treated with Alexa Fluor^®^ 488-conjugated goat anti-rabbit or mouse or 546-conjugated goat anti-rabbit or mouse secondary antibodies at 1:200 or 1:250 (Thermo Scientific, Waltham, MA, USA) for 1 h at RT. Nuclei were then stained with DAPI for 30 min at RT, and the cells were observed under a K1-Fluo confocal laser scanning microscope.

### Immunoprecipitation analysis

VSMC lysates were incubated with anti-PGC-1α or anti-rabbit IgG (Santa Cruz, Delaware, CA, USA) antibodies overnight at 4 °C, as described previously^21^. Protein G beads (Invitrogen, Carlsbad, CA, USA) were added to the immunocomplexes and incubated for 4 h at 4 °C. Unbound proteins were removed by centrifugation, and coimmunoprecipitation products were analyzed by Western blotting.

### Chromatin immunoprecipitation assay

Chromatin immunoprecipitation (ChIP) assays were performed in the usual manner with minor modifications^22^. Briefly, cells were fixed with 1% paraformaldehyde at RT for 10 min, inactivated with 0.125 M glycine, washed twice with ice-cold PBS containing protease inhibitors (1 mM PMSF, 1 mg/mL aprotinin, and 1 mg/mL pepstatin A), scraped, and pelleted by centrifugation at 4 °C. The cells were then resuspended in lysis buffer (1% SDS, 10 mM EDTA, and 50 mM Tris-HCl pH 8.1) containing 100x protease inhibitor, incubated for 10 min on ice, and sonicated eight times to shear DNA (a pulse of 20 s/rest of 30 s) on ice. After sonication, the lysate was centrifuged at 13,000 rpm for 10 min at 4 °C. The supernatant was diluted with ChIP dilution buffer (1% SDS, 1% Triton X-100, 2 mM EDTA, 20 mM Tris-HCl pH 7.4, 150 mM NaCl), and PGC-1α antibody was added and incubated overnight at 4 °C with rotation. The immunocomplex was collected using protein G agarose beads and washed with low-salt washing buffer (0.1% SDS, 1% Triton X-100, 2 mM EDTA, 200 mM Tris-HCl (pH 8.1), and 150 mM NaCl), high-salt buffer (0% SDS, 1% Triton X-100, 2 mM EDTA, 20 mM Tris-HCl (pH 7.4), and 500 mM NaCl), LiCl washing buffer (250 mM LiCl, 1% NP40, 1% sodium deoxycholate, 1 mM EDTA, and 10 mM Tris-HCl (pH 7.4)), and then 1× TE buffer (10 mM Tris-HCl and 1 mM EDTA (pH 8.0)). The immunocomplex was eluted using elution buffer (1% SDS, 100 mM NaHCO_3_), and the cross-links were reversed by heating at 65 °C overnight. Following cross-link reversal, the immunocomplexes were treated with 500 mM EDTA, 1 M Tris-HCl (pH 6.5), and 20 mg/ml proteinase K and incubated for 1 h at 45 °C. Purified DNA was amplified using the following primers:

TERT F 5’-GGTTTTTGAGGGTGAGGGTGAGGGTGAGGGTGAGGG-3’

TERT R 5’-TCCCGACTATCCCTATCCCTATCCCTATCCCTATCCCTA- 3’

When indicated, bound fragments were amplified by PCR (30 cycles of 30 s at 94 °C, 30 s at 60 °C, and 1 min at 72 °C) and visualized by electrophoresing in 1% agarose gel and ethidium bromide staining.

### Real-time measurements of telomerase activity in vitro and in vivo

A telomeric repeat amplification protocol (TRAP) assay based on quantitative PCR (qPCR) was used to determine telomerase activity. The assay was performed using a telomerase detection kit according to the manufacturer’s instructions (Merck Millipore, S7700; MA, USA). Briefly, after treatment, cultured cell pellets were lysed with 1× CHAPS lysis buffer for 30 min on ice. Subsequently, the samples were centrifuged at 13,000×*g* for 20 min at 4 °C, after which the supernatants were collected. The indicated amounts of samples were mixed with 5 μl of TRAP buffer, 1 μl of TS primer (5’-AATCCGTCGAGCAGAGTT- 3’), 1 μl of TRAP primer mix, 1 μl of dNTPs, 0.4 μl of Taq DNA polymerase, 39.6 μl of DEPC-treated H_2_O, and 2 μl of cell extract (0.5 μg), for a total volume of 50 μl. The solution was incubated at 30 °C for 30 min, and then, qPCR was performed using 1 μl of master mixed solution, 1 μl of ACX Primer (5′-GCGCGGCTTACCCTTACCCTTACCCTAACC-3′), and SYBR® Select Master Mix (Enzynomics, Seoul, Korea) and adjusted to a total volume of 20 μl using DEPC-treated H_2_O. The running procedure involved incubation for 10 min at 95 °C and 40 PCR cycles of 10 s at 95 °C, 15 s at 60 °C, and 15 s at 72 °C in a Bio-Rad real-time system (CA, USA). In vivo TRAP activity was also determined by qPCR. In brief, aortic tissue samples were rinsed with ice-1× CHAPS lysis buffer and then homogenized with 100 μl of 1× CHAPS lysis buffer. After 30 min of incubation on ice, the lysate was centrifuged at 13,000×*g* for 20 min at 4 °C. qPCR was performed using the same method used in vitro.

### Telomere PNA-FISH (fluorescence in situ hybridization)

FISH is a cytogenetic technique used to detect and localize the presence or absence of specific DNA sequences on chromosomes^23^. Briefly, VSMCs grown on coverslips were fixed for 30 min on ice in 4% paraformaldehyde. After the cells were washed three times with 1× PBS, they were dehydrated with 70, 85, and 100% ethanol and dried for 15 min at RT. The samples were then treated with a FITC-labeled (CCC TAA)^3^ probe (0.5 μg/ml) (Panagene, Korea), denatured for 5 min at 80 °C, incubated in a wet chamber for 2 h at 37 °C, and washed three times with washing solution (60% formamide, 20 mM Tris pH 7.4). Finally, the cells were stained with DAPI and observed under a K1-Fluo confocal laser scanning microscope.

### In vitro telomere length analysis

Relative telomere lengths were determined by quantitative real-time PCR (qPCR)^24^. In brief, total RNA was isolated from VSMCs using TRIzol reagent (Invitrogen, Carlsbad, CA, USA). RNA concentrations were quantified using a NanoDrop^TM^ 1000 spectrophotometer (Thermo Fisher Scientific, Massachusetts, MA, USA), and cDNA was prepared from total RNA using the SuperScript III First-Strand Synthesis System (Invitrogen, Carlsbad, CA, USA). qPCR was performed using SYBR^®^ Select Master Mix (Enzynomics, Seoul, Korea) and a Bio-Rad real-time system. The processing parameters used were as follows: 95 °C for 10 s, followed by 40 cycles of 15 s at 60 °C and 15 s at 72 °C. The primers used were Tel‐F 5′-GGTTTTTGAGGGTGAGGGTGAGGGTGAGGGTGAGGG‐3′ and Tel‐R 5′‐TCCCGACTATCCCTATCCCTATCCCTATCCCTATCCCTA‐3′ and AT1 rat‐F 5′‐ACGTGTTCTCAGCATCGACCGCTACC‐3′ and AT1 rat‐R 5′‐AGAATGATAAGGAAAGGGAACAAGAAGCCC‐3′ (Bioneer, Daejeon, Korea). The quantity of telomeric DNA was normalized to that of AT1^25^. All samples were run in triplicate. Telomere lengths were calculated using the delta Ct (∆Ct) average Tel DNA and nDNA Ct values (∆Ct = Ct_Tel_ -Ct_AT1_). Relative telomere lengths were calculated using the 2^−∆∆Ct^ method.

### Transfection of siRNA

VSMCs were transfected with control siRNA or siRNA against TERT using Lipofectamine 2000 Reagent (Invitrogen, Carlsbad, CA, USA). Con siRNA and TERT siRNA were purchased from Santa Cruz (Delaware, CA, USA). For knockdown, cells were transfected with 10 μM of each siRNA. Lipofectamine 2000 was removed by placing the cells in a fresh medium containing 10% FBS. Knockdown was confirmed 48 h after transfection before subsequent treatment.

### Gelatin zymography

Extracellular medium from ~100 to 120% confluent VSMCs was used to assess gelatinase activity. The concentrated medium was electrophoretically separated on a 7.5% SDS‒polyacrylamide gel containing 0.4% gelatin (Sigma, St. Louis, MO, USA). The gel was then washed with 2.5% Triton X-100 containing wash buffer, activated in an incubator at 37 °C, and stained with 0.1% Coomassie brilliant blue R-250 (Sigma, St. Louis, MO, USA). Clear zones against a blue background indicated gelatinolytic activity.

### Animal tissues and experiments

All animal studies were approved by the Institutional Animal Care and Use Committee of the College of Medicine, Yeungnam University (YUMC-AEC2022-028). ApoE knockout (KO) mice were kindly donated by Jae-Ryong Kim (Yeungnam University, Daegu, Republic of Korea). Male mice (8 weeks old) were used. The ApoE KO mice had the same genetic background as the C57BL/6J mice. To test the effects of Met on atherosclerotic lesion formation in ApoE KO mice, 42 mice were randomly and equally divided into three groups (each, *n* = 14): the control group (ApoE WT), *n* = 14; the high-fat diet (HFD) group, *n* = 14; and the HFD + Met group, *n* = 14. ApoE WT mice were fed a regular diet for 16 weeks. The HFD-fed mice were fed a high-fat diet containing 35% fat, 20% protein and 45% carbohydrates (Research Diets Inc., New Brunswick, NJ, USA) for 16 weeks. The HFD+Met mice were treated with Met (200 mg/kg/day) by oral administration for 12 weeks after starting 4 weeks of HFD feeding. Mice in the control and model groups were administered an equivalent volume of DW. During the experiment, food intake was monitored, and body weights were measured weekly. The formation of atherosclerotic lesions in the aorta and aortic sinuses was analyzed immediately after sacrifice. Protein samples of whole aortas were obtained from all three groups by lysing samples in RIPA lysis buffer supplemented with 0.01 mM protease inhibitor cocktail (PIC), incubating on ice for 10 min, and centrifuging at 13,000 rpm for 20 min at 4 °C. The protein concentrations in the supernatants were determined using a Bradford assay (Bio-Rad Lab, Hercules, CA, USA). Immunofluorescence analysis was performed using 10% formalin (Sigma, St. Louis, MO, USA)-fixed, paraffin-embedded tissue sections. Whole aortic sections were embedded in paraffin, sectioned at 5 μm, and stained with TERT and α-SMA antibodies or BODIPY probes. Nuclei were stained with DAPI (Invitrogen, Carlsbad, CA, USA). Images were obtained using a K1-Fluo confocal laser scanning microscope at 600×. The slides were stained with hematoxylin and eosin (H&E, Sigma, St. Louis, MO, USA) to assess aortic plaque necrosis and with picrosirius red (Abcam, Cambridge, UK) or Masson’s trichrome (Sigma, St. Louis, MO, USA) to analyze the collagen content in the aortic fibrous caps.

### Plasma lipid measurement

Blood samples were obtained from eyes before sacrifice and centrifuged at 3000 rpm for 5 min, and plasma levels of total cholesterol, triglycerides, LDL-C, and C-reactive protein (CRP) were determined in Laboratory Medicine at Yeungnam University Hospital (Daegu, Republic of Korea) using standard protocols.

### Body composition analysis using DEXA (in vivo dual-energy X-ray absorptiometry)

After 16 weeks on the experimental diet, all mice were subjected to dual-energy X-ray absorptiometry (DEXA, InAlyzer™ MEDIKORS, Korea), which uses low-dose X-ray exposure to determine body fat (g) content with a high degree of precision. The system was calibrated according to the manufacturer’s instructions, and the data analysis was performed using on-board software.

### Atherosclerotic lesion analysis in whole aortas and aortic sinuses

Mice were sacrificed and perfused with ice-cold PBS, and aortic tissues and hearts were harvested. For en face staining, aortas were opened longitudinally and subjected to SAβG activity or oil red O (ORO, Sigma, St. Louis, MO, USA) staining^26^. In addition, whole aortic tissues were subjected to SAβG activity or ORO staining. The extent of atherosclerotic lesions in the aorta and aortic sinuses was analyzed using ImageJ.

### Telomere length analysis in vivo

Relative telomere lengths were determined by qPCR. In brief, total DNA was isolated from aorta tissues using a HelixAmp^TM^ direct PCR kit (NanoHelx Co., Ltd., Daejeon, South Korea). DNA concentrations were quantified using a NanoDrop^TM^ 1000 spectrophotometer (Thermo Fisher Scientific, Massachusetts, MA, USA). qPCR was performed using the same method used in vitro.

### Statistical analysis

For in vitro and in vivo experiments, at least three independent experiments were performed. The results are presented as the means ± SEs. The significance of differences was determined using the unpaired two-tailed Student’s *t*-test and one-way ANOVA or two-way ANOVA followed by Tukey’s post hoc test in GraphPad Prism 8.0.

## Results

### Metformin suppresses oleic acid-induced atherosclerotic and senescent phenotypes in vascular smooth muscle cells (VSMCs)

**Fig. 1:**
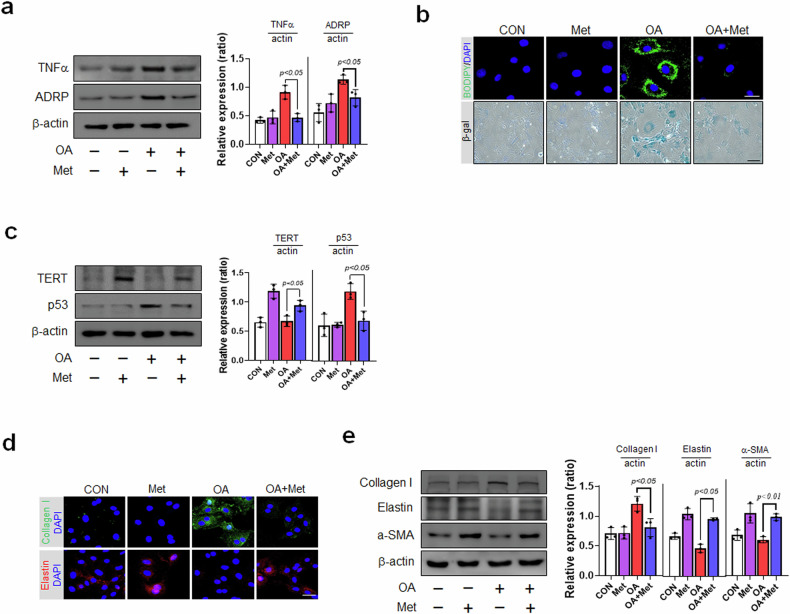
Effects of metformin (Met) on the pathogenesis of oleic acid-induced atherosclerosis and senescent phenotypes. **a** VSMCs were pretreated with oleic acid (OA, 200 μM/24 h) and then incubated with Met (2 mM/1 h). Protein expression levels were analyzed by Western blotting. **b** After treatment with OA and Met, cellular senescence was examined by SA-β-gal staining (blue) (x200, scale bar = 100 μm), followed by BODIPY493/503-based lipid staining (green) (scale bar = 30 μm). **c** Cells were subjected to Western blot analysis using anti-TNF-α and anti-ADRP antibodies. **d** Images of immunofluorescence (IF) staining produced by collagen I antibody (green), elastin (red), and DAPI (blue) of synthetic and contractile VSMCs showing differences in cell morphology (scale bar = 30 μm). **e** Western blot analysis of contractile (elastin and α-SMA) and synthetic proteins (collagen I) in OA- or Met-treated VSMCs. Representative results are shown, and the values are the means ± SEs of three independent experiments.

The effects of Met on the oleic acid (OA)-induced atherosclerotic and senescent phenotypes of vascular smooth muscle cells (VSMCs) were investigated to elucidate the roles of Met in the pathogenesis of atherosclerosis. Western blotting revealed that the protein levels of TNF-α and ADRP (a lipid droplet (LD) marker) were increased by OA, whereas Met reduced these increases (Fig. 1a). We also measured cellular lipid accumulation using the BODIPY probe, which showed that OA treatment caused lipid accumulation in the cytosol and that Met reduced this accumulation (Fig. 1b). Senescent VSMCs are present in aged vessels and atherosclerotic plaques^27^, and we observed that Met downregulated the levels of TERT and p53, which are markers of cellular senescence^28,29^, in OA-induced atherosclerotic and senescent VSMCs (Fig. 1c). We also examined the expression of senescence markers to better understand the mechanisms responsible for the effects of Met on OA-induced VSMC atherosclerotic and senescent phenotypes. When we stained OA-loaded VSMCs with SA-β-gal (senescence-associated beta-galactosidase), the most commonly used biomarker of cellular senescence^30^, OA treatment increased the number of SA- β-gal-positive cells (Fig. 1b), whereas subsequent treatment with Met reduced this increase. VSMC phenotypic switching is known to participate in the pathogenesis of atherosclerosis^31^. As demonstrated by immunofluorescent analysis (IF) (Fig. 1d), the levels of collagen I, a marker of synthetic VSMCs, were significantly decreased by Met in OA-treated VSMCs, while the levels of elastin, a marker of contractile VSMCs, were increased by Met treatment. Therefore, we next investigated whether Met influenced the phenotypic switching of VSMCs by examining the expression of critical markers. OA-loaded VSMCs expressed more collagen I than did Met-treated OA-loaded VSMCs. In contrast, the OA-induced reductions in the expression of elastin and α-SMA, which are markers of contractile VSMCs, were reversed by Met (Fig. 1e). Taken together, these findings suggest that Met significantly suppresses OA-induced atherosclerotic and senescent phenotypes in VSMCs.

### Metformin upregulates TERT protein levels by phosphorylating the AMPK/PGC-1α pathway in OA-treated VSMCs

**Fig. 2:**
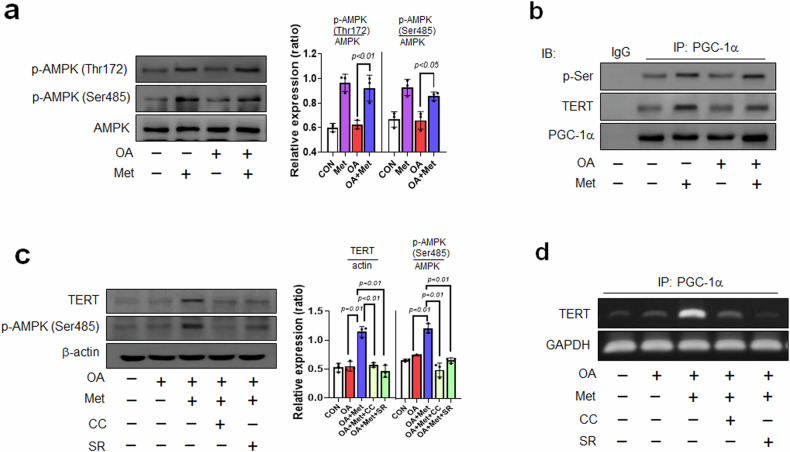
The regulation of TERT expression via the phosphorylation of AMPK and the PGC1-α pathway by metformin (Met). **a** VSMCs were pretreated with OA (200 μM/24 hr) and then incubated with Met (2 mM/1 h). Protein expression levels were analyzed by Western blotting. **b** VSMCs were subjected to immunoprecipitation (IP) using a PGC-1α antibody and then immunoblotted with antibodies recognizing p-Ser (571) and TERT. Total PGC-1α levels in whole-cell lysates were estimated by Western blotting. **c** Western blot analysis of TERT and p-AMPK (Ser485) expression in OA-pretreated VSMCs cotreated with Met, Compound C (CC, 10 μM/1 h) or SR-18292 (SR, 10 μM/24 h). **d** ChIP‒qPCR analysis of TERT with the PGC-1α antibody in OA and Met-treated VSMCs. Representative results are shown, and the values are the means ± SEs of three independent experiments.

The activation of AMP-activated protein kinase (AMPK) by Met is believed to underlie its anti-aging effects^19^. The expression levels of phospho-AMPK (Thr172 and Ser485) were lower in OA-treated VSMCs than in control VSMCs, and this reduction in p-AMPK was reversed by Met treatment (Fig. 2a). PGC-1α acts downstream of AMPK^32^, and the interplay between AMPK and PGC-1α in atherosclerosis involves complex regulatory networks. Previous research has suggested that AMPK activation can stimulate PGC-1α and increase mitochondrial and metabolic functions^33^. We confirmed the effect of Met on the interaction between AMPK and phosphorylated PGC-1α by immunoprecipitation and found that Met-induced phosphorylation of p-Ser on the PGC-1α gene increased the AMPK-mediated phosphorylation of PGC-1α. In addition, this increase in p-Ser expression was almost completely blocked in cells preincubated with OA. Thus, our observations suggest that AMPK activation by Met might activate the PGC-1α protein via direct phosphorylation. We then asked whether the activation of AMPK/PGC-1α by Met affects the expression level of telomerase reverse transcriptase (TERT) and maintains telomere length^34^. As expected, TERT levels were increased by Met, and this increase was blocked by OA pretreatment (Fig. 2b). Therefore, we investigated whether TERT regulation directly contributes to the AMPK/PGC-1α pathway using inhibitors of AMPK and PGC-1α via Western blot and chromatin immunoprecipitation (ChIP) assays. Pretreatment with Compound C (CC, a specific inhibitor of AMPK^35^) or SR-18292 (SR, an inhibitor of PGC-1α^36^) reduced TERT at the protein (Fig. 2c) and DNA (Fig. 2d) levels despite Met treatment in OA-treated VSMCs. Collectively, these results show that Met suppresses atherosclerotic and senescent phenotypes, possibly through the AMPK/PGC-1α/TERT signaling cascade, in OA-treated VSMCs.

### Metformin maintains telomere function and length in OA-treated VSMCs

**Fig. 3:**
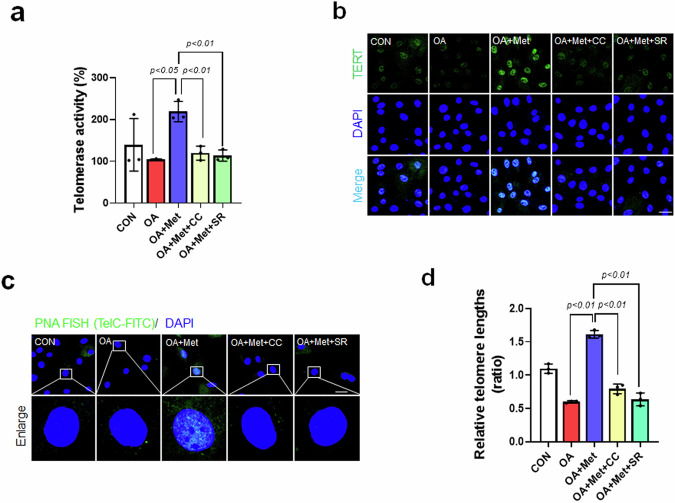
Met-induced upregulation of TERT expression and telomerase activity. **a** After treatment with OA, Met, and CC or SR-18292, telomerase activity was assessed using a TRAP (telomeric repeat amplification) assay. **b** VSMC phenotypes were determined by indirect immunofluorescence staining with TERT (green) (1:200) and DAPI (blue) antibodies (scale bar = 30 μm). **c** Representative images of telomere immunofluorescence-telomere FISH (IF-FISH, green) in VSMCs. Nuclei were stained with DAPI (blue) (scale bar = 30 μm). **d** VSMCs were stimulated with OA and treated with Met and CC or SR-18292. Relative telomere lengths were determined by qPCR. Representative results are shown, and the values are the means ± SEs of three independent experiments.

Telomeres have been proposed to be biological age biomarkers and risk factors for age-related diseases^37^. We found that telomerase activity was significantly induced by Met in OA-treated cells but was reduced by CC or SR (Fig. 3a). Met was also associated with anti-aging and elevated TERT levels^38^. Immunofluorescence analysis revealed that the expression of TERT in the nucleus was markedly increased by Met in OA-treated VSMCs but decreased by CC or SR (Fig. 3b). Telomeres shorten during cell aging and division, and shorter telomeres may cause telomere fusion, inducing genomic instability. Telomere dysfunction can be visualized using a PNA-FISH probe^39^. Therefore, we used a PNA telomere FISH method to visualize telomere signals. PNA telomeres in nuclei were markedly increased by Met in OA-treated VSMCs, but the increase in telomeres was blocked by CC or SR, and decreases in telomerase activity and TERT expression were attributed to reduced telomere signals (Fig. 3c). Furthermore, decreased telomere length has been associated with atherosclerosis^40^, and our qPCR analysis showed that telomere length was shortened in OA-treated VSMCs but not in OA- or Met-treated VSMCs (Fig. 3d). However, treatment with CC or SR did not restore telomere length despite treatment with Met. These observations indicate that Met regulates telomere function and length.

### siRNA-mediated knockdown of TERT accelerates OA-induced atherosclerotic pathogenesis and senescent phenotypes in VSMCs regardless of metformin treatment

**Fig. 4:**
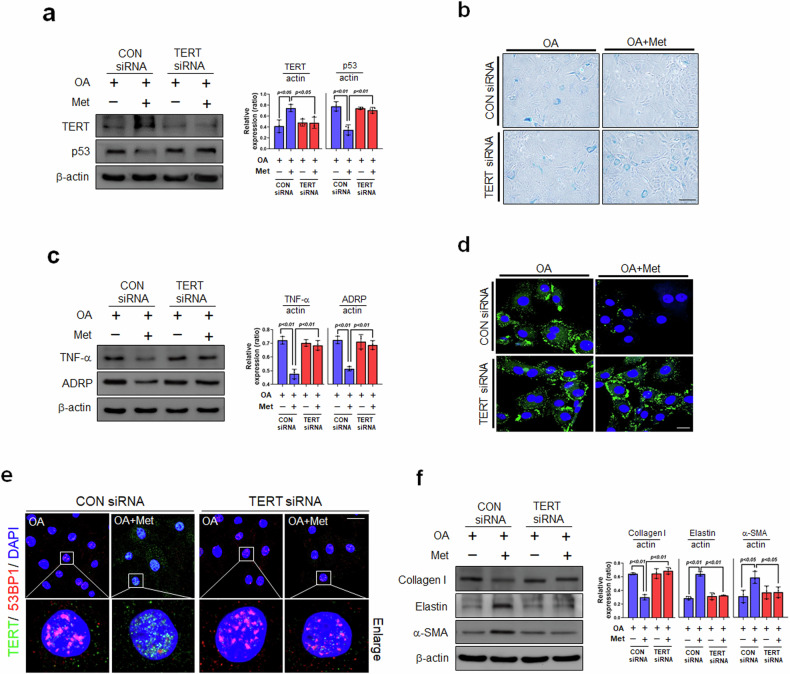
Effect of Met following TERT knockdown during the OA-induced pathogenesis of atherosclerosis and on senescent phenotypes. **a** VSMCs were transfected with control siRNA or TERT siRNA (10 μM) for 48 h, pretreated with 200 μM OA for 24 h, and incubated with 2 mM Met for 1 h, after which the protein levels were analyzed by Western blotting. **b** Cellular senescence was assessed by SA-β-gal staining and bright-field microscopy (200x, scale bar = 100 μm). **c** After transfection, the protein levels were analyzed by Western blotting with TNF-α and ADRP antibodies and **d** the cells were stained with BODIPY493/503 (a green lipid stain) (scale bar = 30 μm). **e** After transfection, colocalization of TERT (green) and 53BP1 foci (red) was observed by confocal microscopy. Nuclei were stained with DAPI (blue). Magnified views of merged images showing details of the colocalization are shown in the lower series of panels (scale bar = 30 μm). **f** Western blotting analysis of contractile (elastin and α−SMA) and synthetic proteins (collagen I) in VSMCs. Representative results are shown, and the values are the means ± SEs of three independent experiments.

To clarify the effect of TERT on atherosclerosis-associated phenotypes in OA-treated VSMCs, we transfected TERT siRNA into VSMCs. Western blotting confirmed the successful knockdown of TERT (Fig. 4a). We found that TERT knockdown induced senescent phenotypes regardless of Met treatment, as evidenced by the induction of p53 (a cellular senescence marker) by Western blotting (Fig. 4a) and the induction of SA-β-gal (Fig. 4b) compared with those in the con siRNA+OA+Met group. We also found that TERT knockdown increased atherosclerosis-associated phenotypes, as indicated by increased TNF-α and ADRP protein levels (Fig. 4c) and increased cellular lipid accumulation, as determined using the BODIPY probe (Fig. 4d), compared with those in the con siRNA+OA+Met group. In addition, immunofluorescence colocalization experiments revealed that TERT fluorescence intensity was weaker in the nuclei of TERT siRNA+OA+Met cells than in those of con siRNA+OA+Met cells, while the number of 53BP1 foci, a DNA damage marker of the aging process^41^, was significantly greater in the nuclei of TERT siRNA+OA+Met cells (Fig. 4e). The switching phenotypes of senescent VSMCs, which were positively correlated with the pathogenesis of atherosclerosis, showed greater collagen I expression in the TERT siRNA+OA+Met group than in the con siRNA+OA+Met group. In contrast, the changes in the expression levels of elastin and α-SMA were not reversed by Met treatment in the TERT siRNA+OA cells compared with those in the con siRNA+OA+Met cells (Fig. 4f). Taken together, these results indicate that TERT knockdown leads to OA-induced atherosclerotic and senescent phenotypes regardless of Met treatment.

### Knockdown of TERT by siRNA increases OA-induced inflammatory responses and senescence-associated secretory phenotypes in VSMCs

**Fig. 5:**
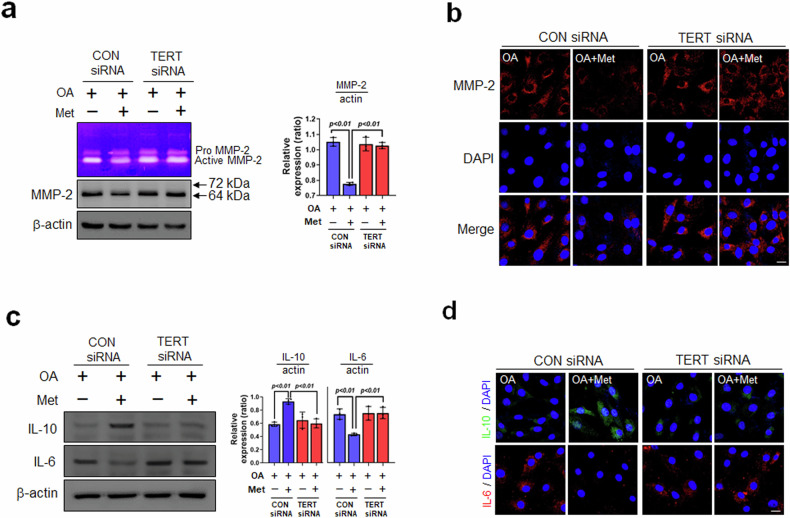
Effect of metformin following TERT knockdown on the OA-induced inflammatory response and senescence-associated secretory phenotype. **a** VSMCs were transfected with TERT siRNA, and then gelatinolytic activity (MMP-2) was determined by gelatin zymography. MMP-2 expression was determined by Western blotting. **b** After transfection, MMP-2 expression was analyzed by immunofluorescence under a confocal microscope (scale bar = 30 μm). **c** To investigate inflammatory cytokines, VSMCs were analyzed by immunoblotting with proinflammatory cytokine (IL-6) and anti-inflammatory cytokine (IL-10) antibodies. **d** IL-10 (green) and IL-6 (red) were analyzed by immunofluorescence under a confocal microscope. Nuclei were stained with DAPI (blue) (scale bar = 30 μm). Representative results are shown, and the values are the means ± SEs of three independent experiments.

Atherosclerosis is a chronic inflammatory vascular disease driven by diverse risk factors^42^. To confirm the role of TERT in OA-induced inflammatory responses and senescence-associated secretory phenotypes (SASPs) of VSMCs under the regulation of Met, we confirmed SASPs in VSMCs transfected with TERT siRNA. Met-induced attenuation of the activity and expression of MMP-2 was significantly enhanced in cells transfected with TERT siRNA (Fig. 5a). IF assays showed that MMP-2 fluorescence intensities were significantly greater in the cytosol of TERT siRNA-transfected cells than in those of the con siRNA+OA+Met group, regardless of Met treatment (Fig. 5b). In addition, we investigated the effects of Met on IL-10 and IL-6 (anti-inflammatory cytokines and proinflammatory cytokines, respectively)^43^ in TERT siRNA-transfected VSMCs. As shown in Fig. 5c, d, Western blotting and IF revealed that the protein expression levels of IL-10 and IL-6 were significantly decreased and increased, respectively, by TERT knockdown regardless of Met treatment. These results demonstrate that TERT knockdown increased OA-induced inflammatory responses and SASPs regardless of Met treatment.

### Metformin suppresses high-fat diet (HFD)-induced atherosclerotic plaque in ApoE knockout (KO) mice

**Fig. 6:**
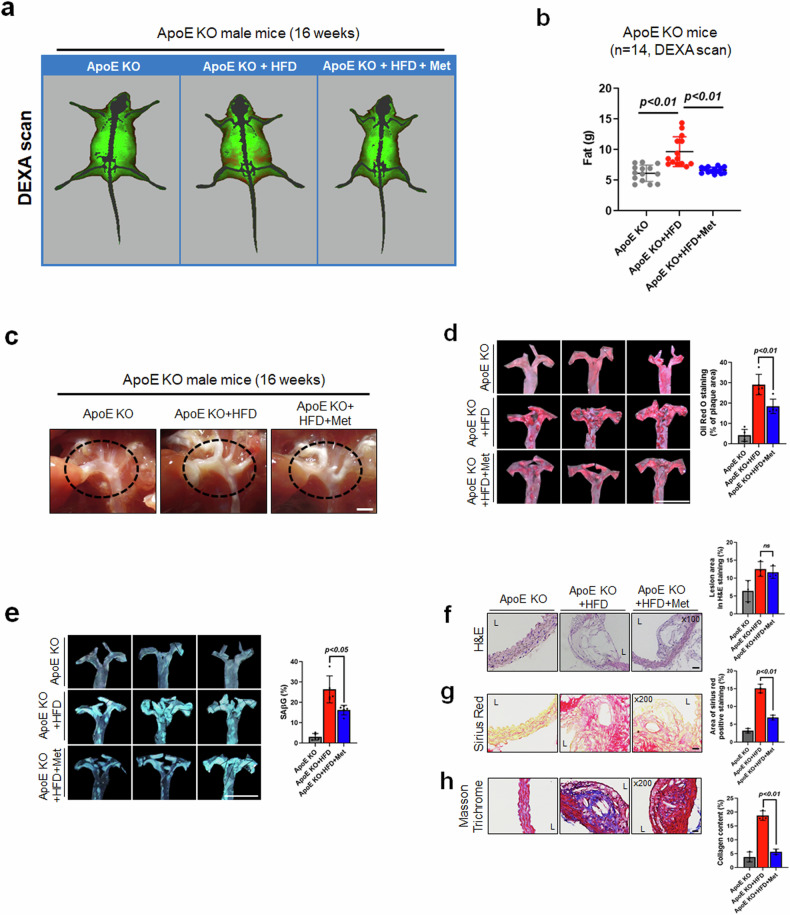
Effect of metformin on advanced atherosclerotic plaques in ApoE KO mice. **a**, **b** DEXA (dual-energy X-ray absorptiometry) images of whole mouse body compositions in the ApoE KO, ApoE, KO + high-fat diet (HFD), and ApoE KO + HFD + Met groups. Changes in total body and abdominal fat density levels. The results are expressed as the means ± SEs of 14 mice. **c** Stereomicroscopic photographs of aortic arches in the ApoE KO, ApoE KO + HFD, and ApoE KO + HFD + Met groups. The dotted circles indicate the region of the aortic arch with a prominent lesion (scale bar = 2 mm). **d** En face oil red O (red) image revealing the ascending region of whole aortas from mice fed an HFD for 16 weeks and the density of the ORO-positive area in en face images (*n* = 4, Scale bar = 5 mm). **e** En face SAβG staining (blue) of the ascending region in whole aortas (*n* = 4, Scale bar = 5 mm). **f** H&E staining was conducted to determine the volumes of atherosclerotic lesions in the aortic sinuses of ApoE KO mice (*n* = 3, scale bar = 100 μm). **g**, **h** Representative images of aortic sinuses stained for collagen fibers (Sirius red and Masson’s trichrome stain) (*n* = 3, Scale bar = 30 μm). Representative results are shown, and the values are the means ± SEs. NS not significant.

Met is known to ameliorate the pathogenesis of atherosclerosis and senescent phenotypes. Therefore, we investigated whether Met suppresses atherosclerosis in ApoE KO mice that develop atherosclerosis due to impaired clearance of plasma lipoproteins^44^. During treatment, we routinely checked consumption and body weights (data not shown) but observed no differences between the ApoE KO (control group) and animals administered the high-fat diet (HFD) or HFD+Met. After a high-fat diet and Met administration, Met-induced reductions in whole-body fat were observed by DEXA scanning (Fig. 6a). The whole-body fat mass (g) results also demonstrated that the HFD-induced increase in fat mass was significantly reduced by Met (Fig. 6b). Additionally, serum lipid (total cholesterol, triglycerides, and LDL-C) levels were higher in the ApoE KO + HFD mice than in the ApoE KO mice, but lower in ApoE KO + HFD+Met mice than in ApoE KO + HFD mice (Table 1). To measure the inflammatory response in the plasma of each group, we evaluated CRP. The ApoE KO + HFD had significantly higher levels of plasma CRP than ApoE KO + HFD+Met (Supplementary Fig. 1). As expected, Met reduced HFD-induced CRP levels in ApoE KO mice. Next, we analyzed the effect of Met on HFD-induced atherosclerosis in ApoE KO mice. Stereomicroscopic observations of the aortic arches, brachiocephalic arteries, carotid arteries, and descending aortas revealed morphometric differences among the three groups (Fig. 6c). Atherosclerosis was more prominent in the aortic roots and descending aortas of the ApoE KO + HFD group than in those of the ApoE KO + HFD+Met group. However, preferential sites were evident in the ApoE KO group. En face analysis of oil red O (ORO)-stained whole aortas revealed atherosclerotic lesions in the ApoE KO + HFD and ApoE KO + HFD+Met groups, and as a result, the ApoE KO + HFD group showed more prominent ORO staining within ascending aortas, aortic arches, and descending aortas than did the ApoE KO + HFD+Met group (Fig. 6d). We then measured SAβG activity in the aorta en face and found that SAβG activity in atherosclerotic lesions was lower in the Met-treated ApoE KO + HFD group than in the ApoE KO + HFD group (Fig. 6e). H&E, Sirius red, and Masson’s trichrome staining of aortas were used to investigate the role of Met in plaque stability^45^. H&E-stained areas, indicating atherosclerotic lesions, were not different between the ApoE KO + HFD and ApoE KO + HFD+Met groups (Fig. 6f). However, Sirius red and Masson’s trichrome staining revealed that the amount of collagen (an indicator of plaque stability) was markedly lower in the ApoE KO + HFD+Met group than in the ApoE KO + HFD group (Fig. 6g, h). Taken together, our findings suggest that Met can efficiently prevent the progression of atherosclerosis and increase the stability of atherosclerotic plaques.

**Table 1:** Serum lipid levels in ApoE KO mice treated with or without metformin.

	Total Cholesterol (mg/dl)	Triglycerides (mg/dl)	LDL-C (mg/dl)	HDL-C (mg/dl)
ApoE KO	288.9 ± 31.7	69.5 ± 8.2	178.6 ± 16.2	35.6 ± 3.4
ApoE KO + HFD	485.1 ± 43.2	103.9 ± 14.2	304.2 ± 30.4	51.0 ± 2.7
ApoE KO + HFD +Metformin	358.6 ± 12.2	59.4 ± 3.7	235.9 ± 10.9	44.5 ± 1.5

*n* = 14 in each group. The data were expressed as the means ± SEs.

### Metformin-induced functional telomere enhancement attenuates advanced atherosclerotic and senescent phenotypes in high-fat diet-fed ApoE KO mice

**Fig. 7:**
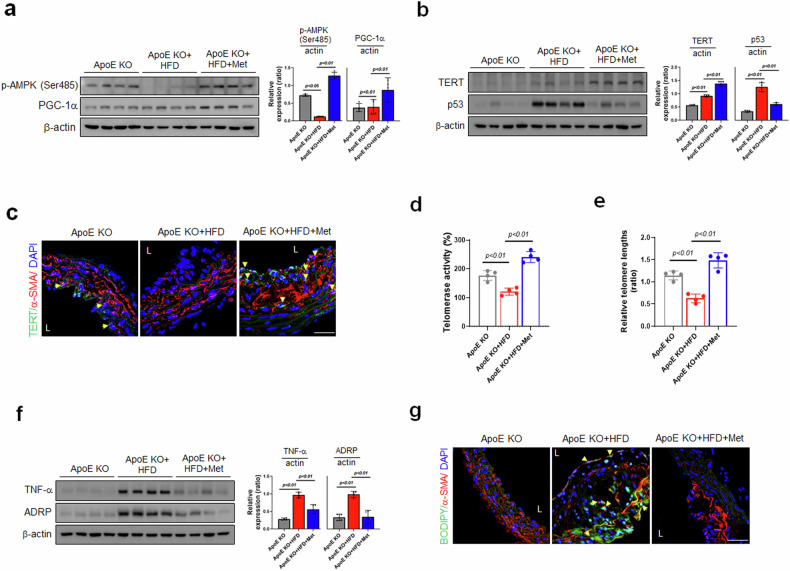
The effects of metformin on TERT expression in advanced atherosclerotic plaques and the senescent phenotypes of high-fat diet-fed ApoE KO mice. **a**, **b** Expression levels of p-AMPK (Ser485), PGC-1α, TERT, p53, and β-actin as determined by Western blotting of aortic tissues from HFD-fed mice (*n* = 4). **c** Immunofluorescence staining and colocalization of TERT (green) and α−SMA (red, a VSMC marker) in fixed tissue sections of atherosclerotic lesions from ApoE KO mice (*n* = 3, Scale bar = 30 μm). **d** After the mice were fed an HFD for 16 weeks, telomerase activity was assessed using a TRAP (telomeric repeat amplification protocol) assay in whole aortas from ApoE KO mice (*n* = 3, same aorta tissue used for DNA analysis). **e** Relative telomere lengths were determined by qPCR (*n* = 3, same aorta tissue for DNA analysis) using DNA samples from whole aortas of ApoE KO mice fed an HFD. **f** Expression levels of TNF-α, ADRP, and α-actin as determined by Western blotting of aortas after HFD consumption (*n* = 4). **g** Immunofluorescence staining and colocalization of BODIPY493/503-based lipid staining (green) and α-SMA (red, a VSMC marker) in fixed tissue sections of atherosclerotic lesions from ApoE KO mice (scale bar = 30 μm). Representative results are shown, and the values are the means ± SEs.

Next, we investigated the mechanisms identified in vitro within the aortas of ApoE KO mice. The expression levels of p-AMPK (Ser485), PGC-1α, and TERT were dramatically greater in the ApoE KO + HFD+Met group than in the ApoE KO + HFD group, whereas the protein level of p53 was markedly lower in the ApoE KO + HFD group than in the ApoE KO + HFD+Met group (Fig. 7a, b). Additionally, IF assays showed that Met administration significantly induced TERT and α-SMA (a marker of smooth muscle cells) in atherosclerotic areas in the ApoE KO + HFD+Met group but not in the ApoE KO + HFD group (Fig. 7c), suggesting that Met attenuates cellular senescence-associated phenotypes and telomere-associated signaling cascades in atherosclerotic lesions. We also investigated whether the Met-induced increase in TERT expression was correlated with telomerase activity and telomere length. Both were significantly increased by Met in ApoE KO + HFD mice (Fig. 7d, e), suggesting that these telomere functions are closely related to the development of atherosclerosis. Consistent with our in vitro results (Fig. 7f), the expression levels of TNF-α and ADRP were significantly lower in the ApoE KO + HFD+Met mice than in the ApoE KO + HFD mice. Furthermore, immunofluorescent analysis revealed that the administration of Met reduced the number of lipid-accumulating VSMCs in atherosclerotic plaques in the ApoE KO + HFD group (Fig. 7g). In summary, these results suggest that Met reduces atherosclerotic plaque formation by enhancing telomere function through the AMPK/PGC-1α pathway in ApoE KO mice.

## Discussion

In this study, we found that Met-induced enhancement of telomere function alleviated the progression of atherosclerosis and attenuated the senescent phenotype of VSMCs. Additionally, our results show that TERT protein levels and telomerase activity control the atherosclerotic and senescent phenotypes of VSMCs. Mechanistically, Met upregulated the protein levels of TERT and promoted telomere function via the phosphorylation of AMPK and the PGC-1α pathway both in vitro and in vivo. Consistent with these findings, genetic inhibition of TERT resulted in a failure of Met to suppress OA-induced atherosclerotic pathogenesis and senescent phenotypes in VSMCs. These results suggest that telomere stabilization by Met protects against atherosclerosis progression via the AMPK-dependent p-PGC-1α pathway.

Atherosclerosis is a complex, chronic cardiovascular disease characterized by the narrowing and hardening of arteries due to the accumulation of fatty deposits, inflammatory cells, and connective tissue within arterial walls^46^. VSMCs play a crucial role in the development and progression of atherosclerosis. In particular, during the progression of atherosclerosis, VSMCs can undergo senescence, that is, a state of irreversible cell cycle arrest and aging-associated functional changes^27,47^. In the context of atherosclerosis, senescent VSMCs are implicated in various pathological processes, including inflammation and senescence-associated secretory phenotypes (SASPs)^48^. However, few studies have evaluated the mechanism linking VSMC senescence and atherosclerosis progression. Here, we found that Met inhibited OA-induced atherosclerotic and senescent phenotypes in VSMCs (Fig. 1).

Met also affects various metabolic pathways, including the AMP-activated protein kinase (AMPK) pathway, which is involved in energy regulation and cellular metabolism^49^, and these effects are thought to play a role in its anti-aging effects. However, despite the similar restorative effects of Met on the decreased expression levels of p-AMPK Thr172 and Ser485 in OA (Fig. 2), we focused on p-AMPK at Ser485 as an indicator of cellular metabolism and senescence because several studies on p-AMPK Thr172 have already been conducted. AMPK phosphorylates more than 100 proteins to modulate metabolic pathways involved in autophagy and lipid, carbohydrate, and protein metabolism^50^. In addition to the catalytic Thr172 activation site, AMPK contains additional phosphorylation sites, including Ser485^51^. Although Thr172 is well known as the main phosphorylation site on AMPK, Thr172, and Ser485 have equivalent effects on cellular senescence. In this study, we observed that Met also phosphorylated both Thr172 and Ser485. Therefore, it is important to clarify the effect of Met on the phosphorylation of AMPK at Ser485, as this finding will reveal a new pathway for the regulation of atherosclerosis. Moreover, emerging evidence suggests that PGC-1α is a major regulator of lifespan and that its interaction with AMPK is important for this function^52^. Vascular senescence is associated with telomeres, mitochondrial dysfunction, and oxidative stress, which suggests that PGC-1α plays a causative role in the pathogenesis of senescence^53^. Here, our data demonstrate that the Met-induced AMPK-dependent phosphorylation of PGC-1α directly increased the expression of TERT in OA-treated VSMCs (Fig. 2). These findings suggest that the Met-induced phosphorylation of AMPK and PGC-1α may be a primary and general modulator of TERT expression, atherosclerosis development, and senescence.

Telomeres play a key role in cardiovascular disease by driving cells into cell cycle arrest and senescence^54^. Telomerase reverse transcriptase (TERT) has long been known for its telomere-lengthening effect^55^. We hypothesized that the link between atherosclerosis and telomeres might be associated with the functional stabilization of telomeres during VSMC senescence. The present study shows that AMPK-dependent p-PGC-1α upregulation is required for the Met-induced upregulation of TERT and telomere activity and length (Fig. 3). However, there appears to be a difference between the restorative effects of Met on AMPK activity and telomere activity, despite the involvement of the AMPK/PGC-1α/TERT signaling pathway. Aging is associated with mitochondrial and nuclear DNA damage; in particular, the accumulation of oxidative damage in mitochondrial DNA (mtDNA) accelerates cellular senescence and shortens telomeres^56^. Among all the organelles, mitochondria are the power plants of eukaryotic cells and also the main site of reactive oxygen species (ROS) production^57^. Substantially, mitochondrial DNA damage caused by ROS can lead to telomere damage in mitochondria. This suggests a connection between mitochondrial dysfunction and telomere damage^52^. Mitochondrial biosynthesis is regulated by the PGC-1 family of coactivators, which consists of PGC-1α and PGC-1β^58^. In addition, energy sensors such as AMPK and SIRT1 have been shown to modulate mitochondrial function^59^. Thus, we propose that telomere instability may affect mitochondrial dysfunction through the DDR. Because ROS induces DNA damage, a persistent DDR is maintained, and this vicious loop locks cells in a deep senescent state^60^. It appears that telomere damage or nuclear DNA damage could affect mitochondrial functions. Because the exact function of mitochondrion-localized TERT activity induced by the AMPK/PGC-1α signaling pathway has not been reported, we suggest that mitochondrial function and telomere stability are conserved by Met-induced p-AMPK Ser485/PGC-1α activation. In contrast, TERT knockdown by siRNA exacerbated telomere dysfunction, OA-induced atherosclerotic phenotypes, and SASPs (Figs. 4, 5). These results indicate that the AMPK/PGC-1α/TERT signaling pathway promoted by Met protects against atherosclerosis and senescence, which suggests that the roles of these signaling pathways in telomere function, cellular aging, and atherosclerosis represent a fertile research area for improving the understanding of these relationships.

This study provides novel evidence of the vascular protective effects of Met on atherosclerosis and some of its underlying mechanisms in nondiabetic ApoE KO mice. Met is one of the most popular oral drugs used to treat hyperglycemia in type 2 diabetes patients^61^, and recently, it was reported that Met has beneficial anti-atherosclerotic^62^ and anti-aging effects^63^. At the molecular level, Met shows anti-aging effects by reducing ATP levels through the activation of AMPK^64^. These data suggest that Met may contribute to improving both healthy lifespans and age-related diseases. However, nonclinical experiments that have reported an anti-aging effect of Met have used higher doses of Met than those used in clinical treatments^65^. The underlying mechanism of Met is not clear, and its anti-aging effects in patients without diabetes have not yet been determined. AMPK activation by Met is known to delay aging. In our study, we confirmed the anti-aging and anti-atherosclerosis effects of Met, an activator of AMPK. Based on our findings, we propose that Met has anti-aging and anti-atherosclerosis effects through the AMPK/PGC-1α/TERT pathway. Although Met is currently being investigated as an anti-aging agent in animal models, our in vitro and in vivo results showed that Met suppresses HFD-induced atherosclerosis by ameliorating VSMC senescence under our experimental conditions. Therefore, our findings suggest that Met may be a promising agent for the prevention and treatment of atherosclerosis, and our findings also demonstrate a novel mechanism and provide new targets for the anti-atherosclerotic effect of Met. We found that Met can effectively modulate the HFD-induced progression of atherosclerosis. First, the reduction in serum lipid profiles (TC, TG, LDL-C) and CRP induced by Met in the ApoE KO + HFD group seemed to be suppressed by the activation of AMPK^66^, which is the main determinant of lipid decreases (Table 1) and inflammatory response reduction (Supplementary Fig. 1). However, in our study, there was no significant difference in plasma HDL levels among the ApoE KO, ApoE KO + HFD and ApoE KO + HFD + Met groups. Moreover, Met administration attenuated HFD-induced atherosclerotic plaque formation (Fig. 6c) and vascular senescence (Fig. 6e) in ApoE KO mice. The reduction of vascular senescence by Met is of particular interest in view of growing evidence suggesting that cellular senescence promotes atherosclerosis^67^. In addition, the present study demonstrated that Met significantly attenuated the formation of atherosclerotic plaques in HFD-fed ApoE KO mice and that the collagen content increased dramatically in the ApoE + HFD group^68^. On the other hand, atherosclerotic plaque areas were similar in the ApoE + HFD and ApoE + HFD+Met groups (Fig. 6). These results show that phenotypic switching by VSMCs during senescence contributes to the accumulation of various types of synthetic VSMCs in atherosclerotic lesions during the pathogenesis of atherosclerosis^31^.

Senescence is characterized by reduced cell telomere shortening and dysfunction^69^. In humans, telomere dysfunction plays a central role and leads to cellular aging and decline^70^. Thus, VSMC senescence is one of the primary risk factors for atherosclerosis. Several studies have investigated the relationship between telomere length and atherosclerosis^71^, but the results of cross-sectional studies have been inconsistent^6^. Therefore, the present study is the first to demonstrate that Met-induced enhancement of telomere function is attenuated in atherosclerosis in ApoE KO mice (Fig. 7).

Overall, we report that Met inhibits the pathogenesis and senescence phenotypes of OA-induced atherosclerosis in VSMCs and that the administration of Met alleviated atherosclerotic plaque formation in the aortas of ApoE KO mice fed a high-fat diet, thus attenuating VSMC senescence-accelerated atherosclerosis. Moreover, Met was found to exert its anti-aging effect on PGC-1α-dependent telomere function and TERT expression by activating AMPK. Our study shows that metformin suppresses senescence-accelerated atherosclerosis in VSMCs and provides evidence supporting the therapeutic value of Met for atherosclerosis.

## Supplementary information

Supplementary Information

## Data Availability

Data will be made available upon receipt of reasonable request.
