# Supplementary material for: Telomere stabilization by metformin mitigates the progression of atherosclerosis via the AMPK-dependent p-PGC-1α pathway
Source: Exp Mol Med. 2024 Sep 2;56(9):1967–79. doi: 10.1038/s12276-024-01297-w (PMC11446938; doi:10.1038/s12276-024-01297-w)
Supplement: Supplementary file 1 — Supplementary Information [file 12276_2024_1297_MOESM1_ESM.pdf]

Supplementary Fig. 1

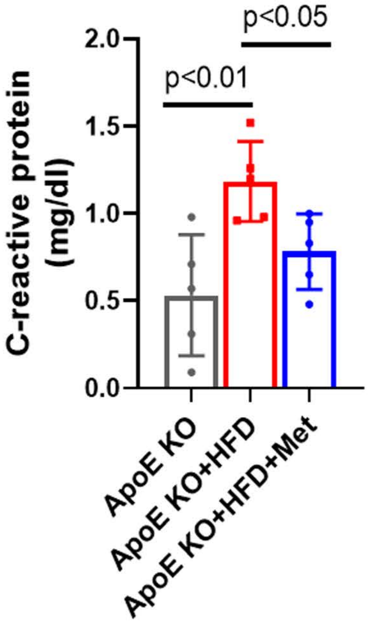

Supplementary Fig. 1 C-reactive protein (CRP) is increased in the plasma of mice fed high fat diet (HFD). Metformin supplementation of high fat fed mice reduced CRP levels. Data are means±SE of *n=5* mice/group.
